# 2,5-Dimethyl-3-(4-methyl­phenyl­sulfon­yl)-1-benzo­furan

**DOI:** 10.1107/S1600536813011392

**Published:** 2013-04-30

**Authors:** Hong Dae Choi, Pil Ja Seo, Uk Lee

**Affiliations:** a Department of Chemistry, Dongeui University, San 24 Kaya-dong, Busanjin-gu, Busan 614-714, Republic of Korea; b Department of Chemistry, Pukyong National University, 599-1 Daeyeon 3-dong, Nam-gu, Busan 608-737, Republic of Korea

## Abstract

In the title compound, C_17_H_16_O_3_S, the dihedral angle between the 4-methyl­phenyl ring and the mean plane [r.m.s. deviation = 0.011 (1) Å] of the benzo­furan ring system is 71.47 (5)°. In the crystal, mol­ecules are linked by weak C—H⋯O hydrogen bonds, and by slipped π–π inter­actions between the benzo­furan ring systems of neighbouring mol­ecules [centroid–centroid distances = 3.638 (2) and 3.766 (2) Å, inter­planar distances = 3.564 (2) and 3.454 (2) Å, and slippages = 0.730 (2) and 1.501 (2) Å], forming a three-dimensional network.

## Related literature

For background information and the crystal structures of related compounds, see: Choi *et al.* (2010 ▶, 2012 ▶).
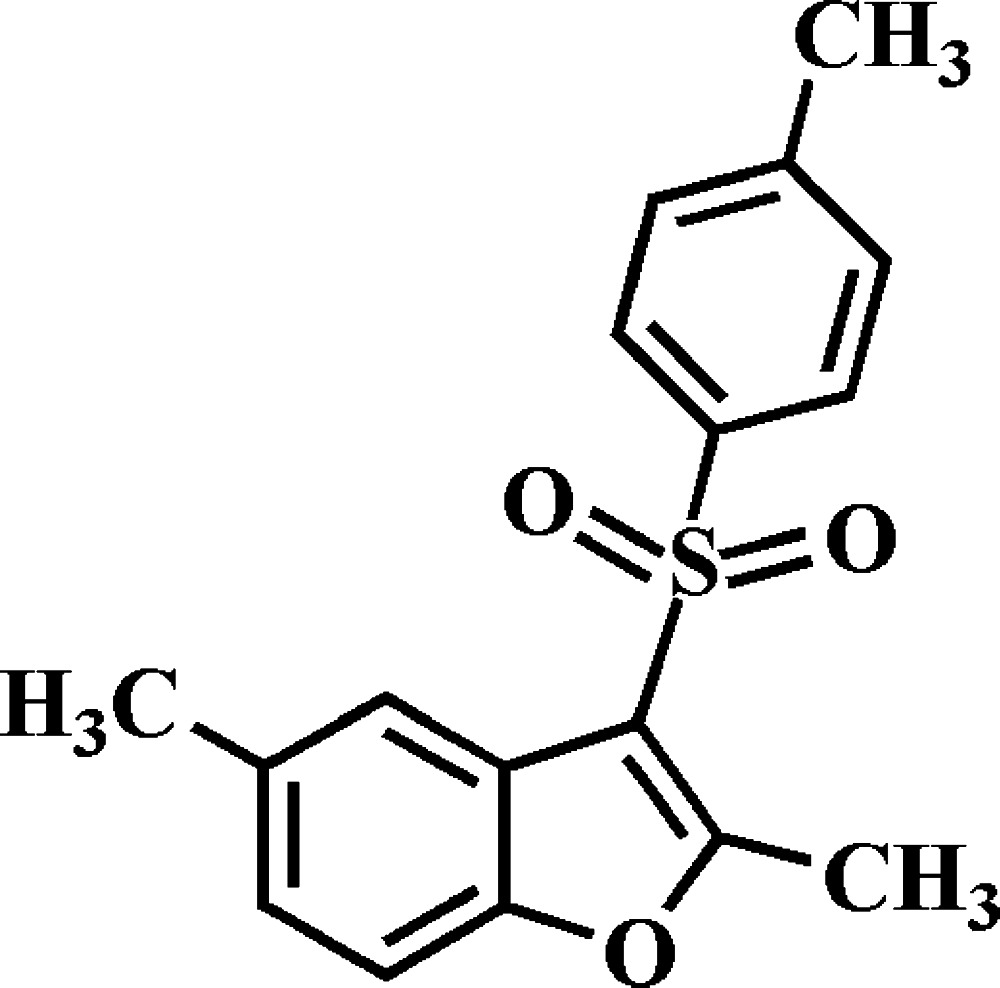


## Experimental

### 

#### Crystal data

C_17_H_16_O_3_S
*M* *_r_* = 300.36
Triclinic, 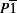

*a* = 7.3481 (3) Å
*b* = 10.2285 (4) Å
*c* = 11.0625 (4) Å
α = 114.134 (2)°
β = 91.056 (2)°
γ = 106.624 (2)°
*V* = 718.30 (5) Å^3^
*Z* = 2
Mo *K*α radiation
μ = 0.23 mm^−1^
*T* = 173 K
0.31 × 0.25 × 0.12 mm

#### Data collection

Bruker SMART APEXII CCD diffractometer
Absorption correction: multi-scan (*SADABS*; Bruker, 2009 ▶) *T*_min_ = 0.684, *T*_max_ = 0.746
13104 measured reflections
3545 independent reflections
2977 reflections with *I* > 2σ(*I*)
*R*_int_ = 0.029

#### Refinement

*R*[*F* ^2^ > 2σ(*F* ^2^)] = 0.041
*wR*(*F* ^2^) = 0.108
*S* = 1.04
3545 reflections
193 parameters
H-atom parameters constrained
Δρ_max_ = 0.39 e Å^−3^
Δρ_min_ = −0.47 e Å^−3^

### 

Data collection: *APEX2* (Bruker, 2009 ▶); cell refinement: *SAINT* (Bruker, 2009 ▶); data reduction: *SAINT*; program(s) used to solve structure: *SHELXS97* (Sheldrick, 2008 ▶); program(s) used to refine structure: *SHELXL97* (Sheldrick, 2008 ▶); molecular graphics: *ORTEP-3* for Windows (Farrugia, 2012 ▶) and *DIAMOND* (Brandenburg, 1998 ▶); software used to prepare material for publication: *SHELXL97*.

## Supplementary Material

Crystal structure: contains datablock(s) global, I. DOI: 10.1107/S1600536813011392/is5267sup1.cif

Structure factors: contains datablock(s) I. DOI: 10.1107/S1600536813011392/is5267Isup2.hkl

Supplementary material file. DOI: 10.1107/S1600536813011392/is5267Isup3.cml

Additional supplementary materials:  crystallographic information; 3D view; checkCIF report

## Figures and Tables

**Table 1:** Hydrogen-bond geometry (Å, °)

*D*—H⋯*A*	*D*—H	H⋯*A*	*D*⋯*A*	*D*—H⋯*A*
C12—H12⋯O2^i^	0.95	2.48	3.224 (2)	135
C16—H16⋯O3^ii^	0.95	2.44	3.301 (2)	151

Symmetry codes: (i) 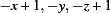; (ii) 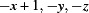.

**Fig. 1.**
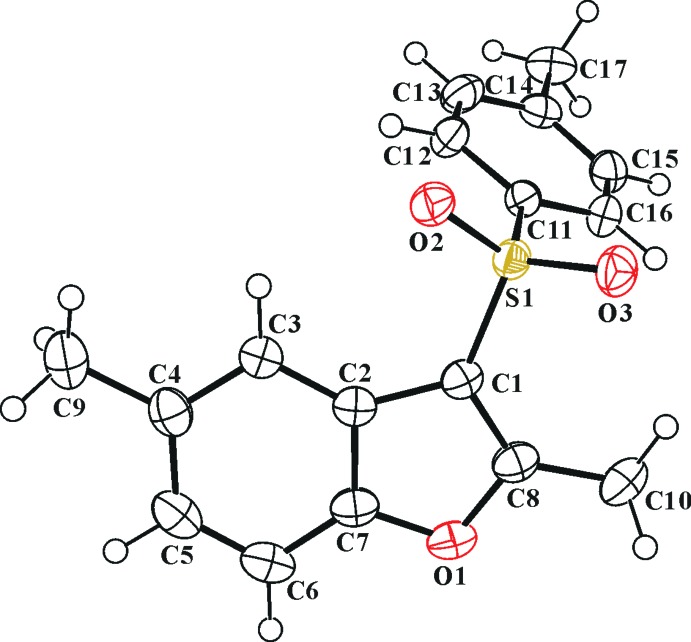
The molecular structure of the title compound with the atom numbering scheme. Displacement ellipsoids are drawn at the 50% probability level. H atoms are presented as small spheres of arbitrary radius.

**Fig. 2.**
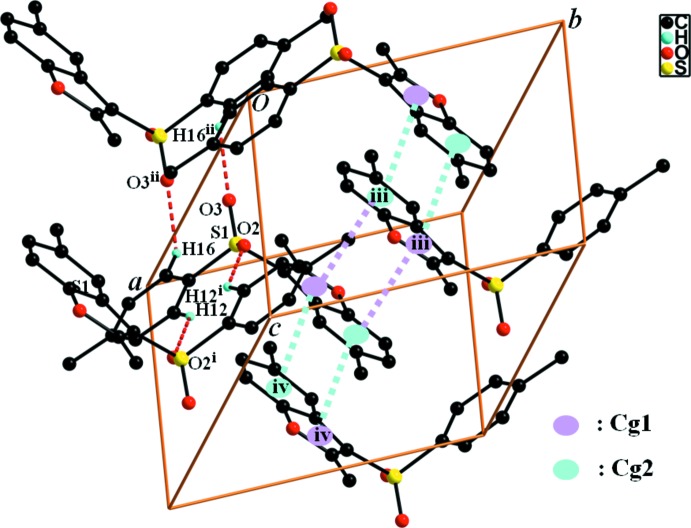
A view of the C—H···O and π–π interactions (dotted lines) in the crystal structure of the title compound. H atoms non-participating in hydrogen-bonding were omitted for clarity. [Symmetry codes: (i) -x + 1, -y, -z + 1; (ii) -x + 1, -y, -z; (iii) -x + 1, -y + 1, -z + 1; (iv) -x + 2, -y + 1, -z + 1.]

## supplementary crystallographic information

### Comment

As a part of our continuing study of 2,5-dimethyl-1-benzofuran derivatives containing 4-chlorophenylsulfonyl (Choi *et al.*, 2010) and 4-methylphenylsulfinyl (Choi *et al.*, 2012) substituents in 3-position, we report herein the crystal structure of the title compound.

In the title molecule (Fig. 1), the benzofuran unit is essentially planar, with a mean deviation of 0.011 (1) Å from the least-squares plane defined by the nine constituent atoms. The dihedral angle between the 4-methylphenyl ring and the mean plane of the benzofuran fragment is 71.47 (5)°. In the crystal structure (Fig. 2), molecules are connected by weak C—H···O hydrogen bonds (Table 1), and by slipped π–π interactions between the furan and benzene rings of neighbouring molecules, with *Cg*1···*Cg*2^iii^ and *Cg*1···*Cg*2^iv^ distances of 3.638 (2) Å & 3.766 (2) Å, and interplanar distances of 3.564 (2) Å & 3.454 (2) Å resulting in slippages of 0.730 (2) Å & 1.501 (2) Å (*Cg*1 and *Cg*2 are the centroids of the C1/C2/C7/O1/C8 furan ring and the C2–C7 benzene ring, respectively).

### Experimental

3-Chloroperoxybenzoic acid (77%, 560 mg, 2.5 mmol) was added in small portions to a stirred solution of 2,5-dimethyl-3-(4-methylphenylsulfanyl)-1-benzofuran (322 mg, 1.2 mmol) in dichloromethane (40 mL) at 273 K. After being stirred at room temperature for 8h, the mixture was washed with saturated sodium bicarbonate solution and the organic layer was separated, dried over magnesium sulfate, filtered and concentrated at reduced pressure. The residue was purified by column chromatography (hexane-ethyl acetate, 4:1 *v*/*v*) to afford the title compound as a colorless solid [yield 73%, m.p. 406–407 K; *R*_f_ = 0.48 (hexane–ethyl acetate, 4:1 *v*/*v*)]. Single crystals suitable for X-ray diffraction were prepared by slow evaporation of a solution of the title compound in acetone at room temperature.

### Refinement

All H atoms were positioned geometrically and refined using a riding model, with C—H = 0.95 Å for aryl and 0.98 Å for methyl H atoms, and with *U*_iso_(H) = 1.2*U*_eq_(C) for aryl and 1.5*U*_eq_(C) for methyl H atoms. The positions of methyl hydrogens were optimized rotationally.

### Crystal data

**Table:**

C_17_H_16_O_3_S	*Z* = 2
*M**_r_* = 300.36	*F*(000) = 316
Triclinic, *P*1	*D*_x_ = 1.389 Mg m^−^^3^
Hall symbol: -P 1	Melting point = 406–407 K
*a* = 7.3481 (3) Å	Mo *K*α radiation, λ = 0.71073 Å
*b* = 10.2285 (4) Å	Cell parameters from 5770 reflections
*c* = 11.0625 (4) Å	θ = 2.3–28.3°
α = 114.134 (2)°	µ = 0.23 mm^−^^1^
β = 91.056 (2)°	*T* = 173 K
γ = 106.624 (2)°	Block, colourless
*V* = 718.30 (5) Å^3^	0.31 × 0.25 × 0.12 mm

### Data collection

**Table:**

Bruker SMART APEXII CCD diffractometer	3545 independent reflections
Radiation source: rotating anode	2977 reflections with *I* > 2σ(*I*)
Graphite multilayer monochromator	*R*_int_ = 0.029
Detector resolution: 10.0 pixels mm^-1^	θ_max_ = 28.3°, θ_min_ = 2.0°
φ and ω scans	*h* = −9→9
Absorption correction: multi-scan (*SADABS*; Bruker, 2009)	*k* = −13→13
*T*_min_ = 0.684, *T*_max_ = 0.746	*l* = −13→14
13104 measured reflections	

### Refinement

**Table:**

Refinement on *F*^2^	Primary atom site location: structure-invariant direct methods
Least-squares matrix: full	Secondary atom site location: difference Fourier map
*R*[*F*^2^ > 2σ(*F*^2^)] = 0.041	Hydrogen site location: difference Fourier map
*wR*(*F*^2^) = 0.108	H-atom parameters constrained
*S* = 1.04	*w* = 1/[σ^2^(*F*_o_^2^) + (0.046*P*)^2^ + 0.3774*P*] where *P* = (*F*_o_^2^ + 2*F*_c_^2^)/3
3545 reflections	(Δ/σ)_max_ < 0.001
193 parameters	Δρ_max_ = 0.39 e Å^−^^3^
0 restraints	Δρ_min_ = −0.47 e Å^−^^3^

### Special details

**Table:**

Geometry. All esds (except the esd in the dihedral angle between two l.s. planes) are estimated using the full covariance matrix. The cell esds are taken into account individually in the estimation of esds in distances, angles and torsion angles; correlations between esds in cell parameters are only used when they are defined by crystal symmetry. An approximate (isotropic) treatment of cell esds is used for estimating esds involving l.s. planes.
Refinement. Refinement of F^2^ against ALL reflections. The weighted R-factor wR and goodness of fit S are based on F^2^, conventional R-factors R are based on F, with F set to zero for negative F^2^. The threshold expression of F^2^ > 2sigma(F^2^) is used only for calculating R-factors(gt) etc. and is not relevant to the choice of reflections for refinement. R-factors based on F^2^ are statistically about twice as large as those based on F, and R- factors based on ALL data will be even larger.

### Fractional atomic coordinates and isotropic or equivalent isotropic displacement parameters (Å^2^)

**Table:**

	*x*	*y*	*z*	*U*_iso_*/*U*_eq_	
S1	0.50135 (6)	0.10427 (4)	0.27607 (4)	0.02799 (12)	
O1	0.81392 (17)	0.52143 (13)	0.36169 (13)	0.0350 (3)	
O2	0.38652 (17)	0.08356 (13)	0.37433 (12)	0.0334 (3)	
O3	0.40868 (17)	0.05919 (14)	0.14334 (12)	0.0371 (3)	
C1	0.6363 (2)	0.29425 (18)	0.34342 (16)	0.0279 (3)	
C2	0.6791 (2)	0.39740 (18)	0.48433 (16)	0.0278 (3)	
C3	0.6357 (2)	0.38832 (19)	0.60305 (16)	0.0306 (3)	
H3	0.5622	0.2948	0.6021	0.037*	
C4	0.7017 (2)	0.5182 (2)	0.72308 (17)	0.0353 (4)	
C5	0.8078 (3)	0.6558 (2)	0.72172 (19)	0.0400 (4)	
H5	0.8498	0.7443	0.8043	0.048*	
C6	0.8531 (3)	0.6672 (2)	0.60581 (19)	0.0383 (4)	
H6	0.9257	0.7607	0.6063	0.046*	
C7	0.7877 (2)	0.53589 (19)	0.48886 (18)	0.0315 (4)	
C8	0.7209 (2)	0.37347 (19)	0.27511 (18)	0.0321 (4)	
C9	0.6603 (3)	0.5108 (2)	0.85310 (19)	0.0485 (5)	
H9A	0.5486	0.4219	0.8355	0.073*	
H9B	0.6332	0.6025	0.9126	0.073*	
H9C	0.7722	0.5030	0.8960	0.073*	
C10	0.7368 (3)	0.3328 (2)	0.13212 (19)	0.0423 (4)	
H10A	0.7052	0.4059	0.1067	0.064*	
H10B	0.6473	0.2312	0.0770	0.064*	
H10C	0.8685	0.3344	0.1178	0.064*	
C11	0.6690 (2)	0.00704 (17)	0.26085 (15)	0.0276 (3)	
C12	0.7065 (3)	−0.0350 (2)	0.36046 (17)	0.0337 (4)	
H12	0.6457	−0.0089	0.4380	0.040*	
C13	0.8334 (3)	−0.1156 (2)	0.34560 (19)	0.0376 (4)	
H13	0.8570	−0.1465	0.4128	0.045*	
C14	0.9267 (2)	−0.15207 (18)	0.23464 (18)	0.0337 (4)	
C15	0.8911 (3)	−0.1045 (2)	0.13838 (17)	0.0361 (4)	
H15	0.9576	−0.1255	0.0634	0.043*	
C16	0.7616 (3)	−0.02729 (19)	0.14912 (16)	0.0337 (4)	
H16	0.7361	0.0020	0.0811	0.040*	
C17	1.0609 (3)	−0.2432 (2)	0.2168 (2)	0.0441 (4)	
H17A	1.1135	−0.2298	0.3047	0.066*	
H17B	1.1661	−0.2089	0.1726	0.066*	
H17C	0.9903	−0.3505	0.1616	0.066*	

### Atomic displacement parameters (Å^2^)

**Table:**

	*U*^11^	*U*^22^	*U*^33^	*U*^12^	*U*^13^	*U*^23^
S1	0.0275 (2)	0.0299 (2)	0.0247 (2)	0.00481 (15)	0.00166 (15)	0.01299 (16)
O1	0.0319 (6)	0.0332 (6)	0.0455 (7)	0.0079 (5)	0.0067 (5)	0.0241 (6)
O2	0.0318 (6)	0.0342 (6)	0.0320 (6)	0.0048 (5)	0.0081 (5)	0.0160 (5)
O3	0.0362 (7)	0.0431 (7)	0.0286 (6)	0.0086 (5)	−0.0019 (5)	0.0155 (5)
C1	0.0249 (7)	0.0302 (8)	0.0310 (8)	0.0082 (6)	0.0034 (6)	0.0159 (6)
C2	0.0234 (7)	0.0286 (8)	0.0334 (8)	0.0098 (6)	0.0036 (6)	0.0142 (6)
C3	0.0289 (8)	0.0304 (8)	0.0324 (8)	0.0097 (6)	0.0042 (6)	0.0134 (7)
C4	0.0335 (9)	0.0373 (9)	0.0333 (9)	0.0161 (7)	0.0040 (7)	0.0105 (7)
C5	0.0375 (9)	0.0315 (9)	0.0428 (10)	0.0139 (7)	−0.0023 (8)	0.0068 (8)
C6	0.0315 (9)	0.0283 (8)	0.0526 (11)	0.0088 (7)	−0.0002 (8)	0.0159 (8)
C7	0.0259 (8)	0.0316 (8)	0.0421 (9)	0.0108 (6)	0.0048 (7)	0.0198 (7)
C8	0.0275 (8)	0.0348 (8)	0.0393 (9)	0.0101 (7)	0.0046 (7)	0.0211 (7)
C9	0.0560 (12)	0.0507 (12)	0.0327 (10)	0.0198 (10)	0.0069 (9)	0.0103 (9)
C10	0.0434 (10)	0.0529 (11)	0.0418 (10)	0.0139 (9)	0.0111 (8)	0.0319 (9)
C11	0.0290 (8)	0.0259 (7)	0.0245 (7)	0.0040 (6)	0.0005 (6)	0.0109 (6)
C12	0.0381 (9)	0.0368 (9)	0.0286 (8)	0.0088 (7)	0.0073 (7)	0.0184 (7)
C13	0.0413 (10)	0.0395 (9)	0.0393 (9)	0.0111 (8)	0.0041 (8)	0.0254 (8)
C14	0.0318 (8)	0.0249 (8)	0.0391 (9)	0.0034 (6)	0.0024 (7)	0.0128 (7)
C15	0.0407 (9)	0.0352 (9)	0.0290 (8)	0.0114 (7)	0.0083 (7)	0.0110 (7)
C16	0.0413 (9)	0.0356 (9)	0.0237 (8)	0.0104 (7)	0.0034 (7)	0.0138 (7)
C17	0.0402 (10)	0.0351 (9)	0.0599 (12)	0.0123 (8)	0.0089 (9)	0.0230 (9)

### Geometric parameters (Å, º)

**Table:**

S1—O2	1.4347 (12)	C9—H9B	0.9800
S1—O3	1.4360 (12)	C9—H9C	0.9800
S1—C1	1.7323 (16)	C10—H10A	0.9800
S1—C11	1.7628 (16)	C10—H10B	0.9800
O1—C8	1.369 (2)	C10—H10C	0.9800
O1—C7	1.376 (2)	C11—C12	1.386 (2)
C1—C8	1.358 (2)	C11—C16	1.388 (2)
C1—C2	1.445 (2)	C12—C13	1.382 (2)
C2—C3	1.390 (2)	C12—H12	0.9500
C2—C7	1.393 (2)	C13—C14	1.386 (3)
C3—C4	1.389 (2)	C13—H13	0.9500
C3—H3	0.9500	C14—C15	1.388 (2)
C4—C5	1.407 (3)	C14—C17	1.504 (2)
C4—C9	1.503 (3)	C15—C16	1.378 (2)
C5—C6	1.373 (3)	C15—H15	0.9500
C5—H5	0.9500	C16—H16	0.9500
C6—C7	1.377 (2)	C17—H17A	0.9800
C6—H6	0.9500	C17—H17B	0.9800
C8—C10	1.478 (2)	C17—H17C	0.9800
C9—H9A	0.9800		
			
O2—S1—O3	119.29 (7)	C4—C9—H9C	109.5
O2—S1—C1	106.76 (7)	H9A—C9—H9C	109.5
O3—S1—C1	109.07 (8)	H9B—C9—H9C	109.5
O2—S1—C11	107.90 (7)	C8—C10—H10A	109.5
O3—S1—C11	107.52 (7)	C8—C10—H10B	109.5
C1—S1—C11	105.50 (7)	H10A—C10—H10B	109.5
C8—O1—C7	107.01 (12)	C8—C10—H10C	109.5
C8—C1—C2	107.56 (14)	H10A—C10—H10C	109.5
C8—C1—S1	126.87 (13)	H10B—C10—H10C	109.5
C2—C1—S1	125.57 (12)	C12—C11—C16	120.60 (16)
C3—C2—C7	119.29 (15)	C12—C11—S1	119.59 (13)
C3—C2—C1	136.09 (15)	C16—C11—S1	119.81 (12)
C7—C2—C1	104.60 (15)	C13—C12—C11	119.20 (16)
C4—C3—C2	118.91 (16)	C13—C12—H12	120.4
C4—C3—H3	120.5	C11—C12—H12	120.4
C2—C3—H3	120.5	C12—C13—C14	121.31 (16)
C3—C4—C5	119.47 (17)	C12—C13—H13	119.3
C3—C4—C9	119.97 (17)	C14—C13—H13	119.3
C5—C4—C9	120.56 (17)	C13—C14—C15	118.30 (16)
C6—C5—C4	122.60 (17)	C13—C14—C17	121.26 (16)
C6—C5—H5	118.7	C15—C14—C17	120.44 (17)
C4—C5—H5	118.7	C16—C15—C14	121.53 (16)
C5—C6—C7	116.39 (17)	C16—C15—H15	119.2
C5—C6—H6	121.8	C14—C15—H15	119.2
C7—C6—H6	121.8	C15—C16—C11	119.01 (15)
O1—C7—C6	126.27 (16)	C15—C16—H16	120.5
O1—C7—C2	110.39 (14)	C11—C16—H16	120.5
C6—C7—C2	123.32 (17)	C14—C17—H17A	109.5
C1—C8—O1	110.43 (15)	C14—C17—H17B	109.5
C1—C8—C10	133.95 (17)	H17A—C17—H17B	109.5
O1—C8—C10	115.60 (15)	C14—C17—H17C	109.5
C4—C9—H9A	109.5	H17A—C17—H17C	109.5
C4—C9—H9B	109.5	H17B—C17—H17C	109.5
H9A—C9—H9B	109.5		
			
O2—S1—C1—C8	−159.43 (14)	C1—C2—C7—C6	177.78 (15)
O3—S1—C1—C8	−29.29 (17)	C2—C1—C8—O1	−0.70 (18)
C11—S1—C1—C8	85.95 (16)	S1—C1—C8—O1	179.36 (11)
O2—S1—C1—C2	20.64 (15)	C2—C1—C8—C10	177.32 (18)
O3—S1—C1—C2	150.78 (13)	S1—C1—C8—C10	−2.6 (3)
C11—S1—C1—C2	−93.98 (14)	C7—O1—C8—C1	0.23 (17)
C8—C1—C2—C3	179.54 (18)	C7—O1—C8—C10	−178.19 (14)
S1—C1—C2—C3	−0.5 (3)	O2—S1—C11—C12	−17.41 (15)
C8—C1—C2—C7	0.87 (17)	O3—S1—C11—C12	−147.28 (13)
S1—C1—C2—C7	−179.19 (12)	C1—S1—C11—C12	96.42 (14)
C7—C2—C3—C4	0.2 (2)	O2—S1—C11—C16	162.29 (13)
C1—C2—C3—C4	−178.31 (17)	O3—S1—C11—C16	32.42 (15)
C2—C3—C4—C5	1.0 (2)	C1—S1—C11—C16	−83.88 (14)
C2—C3—C4—C9	−178.75 (16)	C16—C11—C12—C13	−1.8 (3)
C3—C4—C5—C6	−1.4 (3)	S1—C11—C12—C13	177.88 (13)
C9—C4—C5—C6	178.35 (17)	C11—C12—C13—C14	1.4 (3)
C4—C5—C6—C7	0.5 (3)	C12—C13—C14—C15	0.6 (3)
C8—O1—C7—C6	−178.12 (16)	C12—C13—C14—C17	−178.38 (16)
C8—O1—C7—C2	0.35 (17)	C13—C14—C15—C16	−2.2 (3)
C5—C6—C7—O1	179.09 (15)	C17—C14—C15—C16	176.74 (16)
C5—C6—C7—C2	0.8 (3)	C14—C15—C16—C11	1.8 (3)
C3—C2—C7—O1	−179.69 (13)	C12—C11—C16—C15	0.2 (2)
C1—C2—C7—O1	−0.75 (17)	S1—C11—C16—C15	−179.48 (13)
C3—C2—C7—C6	−1.2 (2)		

### Hydrogen-bond geometry (Å, º)

**Table:**

*D*—H···*A*	*D*—H	H···*A*	*D*···*A*	*D*—H···*A*
C12—H12···O2^i^	0.95	2.48	3.224 (2)	135
C16—H16···O3^ii^	0.95	2.44	3.301 (2)	151

Symmetry codes: (i) −*x*+1, −*y*, −*z*+1; (ii) −*x*+1, −*y*, −*z*.
